# Multi-Dimensional, Short-Timescale Quantification of Parkinson's Disease and Essential Tremor Motor Dysfunction

**DOI:** 10.3389/fneur.2020.00886

**Published:** 2020-09-18

**Authors:** John B. Sanderson, James H. Yu, David D Liu, Daniel Amaya, Peter M. Lauro, Anelyssa D'Abreu, Umer Akbar, Shane Lee, Wael F. Asaad

**Affiliations:** ^1^The Warren Alpert Medical School, Brown University, Providence, RI, United States; ^2^Department of Neurosurgery, Rhode Island Hospital, Providence, RI, United States; ^3^Department of Neuroscience, Brown University, Providence, RI, United States; ^4^Carney Institute for Brain Science, Brown University, Providence, RI, United States; ^5^Norman Prince Neurosciences Institute, Rhode Island Hospital, Providence, RI, United States; ^6^Department of Neurology, Rhode Island Hospital, Providence, RI, United States

**Keywords:** deep brain stimulation, Parkinson's Disease (PD), essential tremor (ET), machine learning, UPDRS, symptom assessment

## Abstract

**Introduction:** Parkinson's disease (PD) is a progressive movement disorder characterized by heterogenous motor dysfunction with fluctuations in severity. Objective, short-timescale characterization of this dysfunction is necessary as therapies become increasingly adaptive.

**Objectives:** This study aims to characterize a novel, naturalistic, and goal-directed tablet-based task and complementary analysis protocol designed to characterize the motor features of PD.

**Methods:** A total of 26 patients with PD and without deep brain stimulation (DBS), 20 control subjects, and eight patients with PD and with DBS completed the task. Eight metrics, each designed to capture an aspect of motor dysfunction in PD, were calculated from 1-second, non-overlapping epochs of the raw positional and pressure data captured during task completion. These metrics were used to generate a classifier using a support vector machine (SVM) model to produce a unifying, scalar “motor error score” (MES). The data generated from these patients with PD were compared to same-day standard clinical assessments. Additionally, these data were compared to analogous data generated from a separate group of 12 patients with essential tremor (ET) to assess the task's specificity for different movement disorders. Finally, an SVM model was generated for each of the eight patients with PD and with DBS to differentiate between their motor dysfunction in the “DBS On” and “DBS Off” stimulation states.

**Results:** The eight metrics calculated from the raw positional and force data captured during task completion were non-redundant. MES generated by the SVM analysis protocol showed a strong correlation with MDS-UPDRS-III scores assigned by movement disorder specialists. Analysis of the relative contributions of each of the eight metrics showed a significant difference between the motor dysfunction of PD and ET. Much of this difference was attributable to the homogenous, tremor-dominant phenotype of ET motor dysfunction. Finally, in individual patients with PD with DBS, task performance and subsequent SVM classification effectively differentiated between the “DBS On” and “DBS Off” stimulation states.

**Conclusion:** This tablet-based task and analysis protocol correlated strongly with expert clinical assessments of PD motor dysfunction. Additionally, the task showed specificity for PD when compared to ET, another common movement disorder. This specificity was driven by the relative heterogeneity of motor dysfunction of PD compared to ET. Finally, the task was able to distinguish between the “DBS On” and “DBS Off” states within single patients with PD. This task provides temporally-precise and specific information about motor dysfunction in at least two movement disorders that could feasibly correlate to neural activity.

## Introduction

Parkinson's disease (PD) is the second-most common neurodegenerative disease worldwide, with an overall prevalence of 0.3 percent (1, 2) and a prevalence of two percent in people above age 70 (3). It is diagnosed clinically based on the presence of bradykinesia and at least one of the following three signs: rest tremor, postural instability, and rigidity. In practice, symptomatology is diverse (4), making comparison of disease severity between patients difficult (5).

Currently, the Movement Disorder Society-Sponsored Revision of the Unified PD Rating Scale (MDS-UPDRS), a rating system developed in 1987 and revised in 2007 (6, 7), remains the standard clinical scale for the evaluation of PD severity (6, 8–12). It consists of five sections, which account for a patient's ability to perform activities of daily living, degree of motor impairment, and alterations in behavior, mood, and cognition. The Motor Examination section (MDS-UPDRS-III) specifically assesses motor impairment, and scoring in this section can alter clinical management (13, 14). The MDS-UPDRS-III consists of 14 subsections, each rated from zero (not present) to four (most severe). Accurate score assignment relies on the experience of the evaluator, and it depends on the patient's medication state and their point in the natural fluctuation of motor dysfunction at the time of evaluation. While it is a useful and validated tool with high inter-rater consistency (8, 9, 11, 15), it cannot provide the immediate, continuous, and temporally precise quantitative data that are required for identifying the neural correlates that dictate increasingly prevalent adaptive and personalized therapies.

The demand for a temporally precise measures of PD motor dysfunction is reflected in the growing body of literature describing technology-based motor assessments. As technology companies, such as Apple Inc., have expanded their health monitoring services, PD has been a focus of early large-scale data gathering studies, such as mPower (16). Many of these approaches, like the one presented in this report, are task-based and require a patient to generate data electively (17–22). Other approaches use background-running software to collect data from a patient's quotidian interactions with their devices (23–26). Still others utilize accelerometers in wearable devices to gather continuous data (27). Some systems evaluate specific domains of motor dysfunction (28, 29), while many, like the one presented in this study, aim for a more comprehensive appraisal. These methods are growing in their acceptance, and researchers are now using improvement as measured by smartphone-based testing as an exploratory endpoint in therapeutic clinical trials (30). While these approaches each have relative advantages and drawbacks, each yields large data sets that will expand our understanding of movement disorders at both a population and an individual level.

Here, we introduce a novel, goal-directed, and naturalistic tablet-based task and a complementary analytic approach to improve upon currently available assessments of PD motor impairment. Specifically, we sought to increase the temporal precision of motor assessment while accounting for the heterogeneity of PD motor dysfunction by using a stylus-mediated “target tracking task” combined with a multidimensional, machine-learning based analysis of multiple movement-derived metrics. We compared patients with PD to non-movement disorder control subjects using this behavioral task and found it to discriminate between these groups with high accuracy at short timescales. This multi-dimensional approach improves upon recently described assessments that rely on fewer metrics (18, 19, 21, 22). Additionally, our approach showed specificity when compared to another movement disorder, essential tremor (ET). Our analysis showed significantly different contributions of each metric to our support vector machine (SVM) classifier in PD and ET. Our SVM-based classification protocol also differentiated between stimulation states in patients with PD with deep brain stimulation (DBS). These results suggest that this objective, multi-dimensional approach to movement disorder assessment can provide information about the motor dysfunction of patients with movement disorders with the temporal precision necessary for correlation to neural activity.

## Methods

### Study Participation

Patients undergoing follow-up care or consultation for neuromodulation therapy for either PD or ET at the Rhode Island Hospital movement disorders clinic between 2017 and 2018 were offered the option to participate in this study. No compensation was provided. To avoid possible confounding due to cognitive impairment, a common feature of advanced PD, only patients who were able to demonstrate a clear understanding of the task were asked to participate.

Approximately age-matched controls (often patients' spouses or partners) also participated in this study. Control subjects were required only to be free of any diagnosed or suspected movement disorder and to have no physical limitation preventing them from seeing the display or appropriately manipulating the stylus.

Subjects agreeing to participate in this study signed informed consent documents and the task was administered in accordance with Rhode Island Hospital human research protocol (Lifespan IRB #263157) and the Declaration of Helsinki. All subject data were de-identified. Two-letter subject identifiers that appear in this report were randomly generated and unrelated to subject initials.

Ultimately, 26 patients with PD and without DBS and 12 patients with ET completed the task. Additionally, 20 control subjects volunteered to participate (Table 1). Patients with PD who participated in the study were significantly older than control subjects (69.69, SD ± 8.61, compared to 58.45, SD ± 10.20, *T*-test, *T* = 3.994, *p* = 0.0002). Patients in the PD group had a mean duration of disease of 7.32 years (SD ± 5.94), compared to a mean duration of 13.46 years in the ET group (SD ± 14.52). Because control subjects were frequently the spouses of participating patients, the distribution of self-identified genders in the patient groups differed from that of the control group, although this difference was only significant in the PD group, in which more males than females participated (Chi-square tests *p*-values: PD = 2.67 × 10^−8^; ET = 0.0866).

**Table 1:** Subject characteristics.

	**PD (Non-DBS)**				**ET**				**Control**		
	**Documented**	**Mean**	**SD**	***p*-value (to control)**	**Documented**	**Mean**	**SD**	***p*-value (to control)**	**Documented**	**Mean**	**SD**
Total	26	-	-	**2.67** **×** **10**^**−8**^	12	-	-	0.087	20	-	-
Men	20	-	-	-	5	-	-	-	6	-	-
Women	6	-	-	-	7	-	-	-	13	-	-
Age	26	69.69	8.62	**0.0002**	12	65.83	12.99	0.084	20	58.45	10.20
Disease duration	26	7.32	5.94	-	12	13.46	14.52	-	-	-	-
Handedness	26	-	-	0.169	12	-	-	**0.0218**	20	-	-
R-handed	25	-	-	-	9	-	-	-	19	-	-
L-handed	1	-	-	-	1	-	-	-	1	-	-
Ambidextrous	0	-	-	-	2	-	-	-	0	-	-
Last meds	23	4.13	4.86	-	5	17.60	14.50	-	-	-	-
Predominant phenotype	26	-	-	-	-	-	-	-	-	-	-
TD	12	-	-	-	-	-	-	-	-	-	-
PGID	11	-	-	-	-	-	-	-	-	-	-
Mixed	3	-	-	-	-	-	-	-	-	-	-
		**PD (DBS)**									
		**Documented**			**Mean**		**SD**		**p-value (to non-DBS PD)**		
Total		8			-		-		**3.56** **×** **10**^**−4**^		
Men		5			-		-		-		
Women		3			-		-		-		
Age		8			60.63		7.09		0.0501		
Disease duration		8			10.75		2.81		0.118		
Handedness		8			-		-		**0.0067**		
R-handed		8			-		-		-		
L-handed		0			-		-		-		
Ambidextrous		0			-		-		-		
Years since implant		8			1.75		1.16		-		
Last meds		8			3.44		2.09		0.574		

*Significant results in bold. Each PD patient was classified as “Tremor-Dominant” (TD), “Postural Instability/Gait Difficulty” (PGID), or “mixed”*.

Patients with PD and without DBS were classified according to their phenotype based on previously described analyses of MDS-UPDRS-III subsection scores (31, 32). Briefly, if the ratio of the average of the “tremor” scores to the average of the “postural instability” and “gait difficulty” scores exceeded 1.5, the patient was considered “tremor-dominant” (TD). If this ratio was between 1 and 1.5, the patient is considered “mixed,” and if the ratio was less than 1, the patient was considered “postural instability/gait difficulty” (PIGD).

An additional eight patients with PD with DBS completed the task. Patients with PD with DBS were not significantly different from patients with PD without DBS in age (62.63, SD ± 7.09, compared to 69.69, SD ± 8.61, *T*-test, *T* = 2.033, *p* = 0.0501), disease duration (10.75, SD ± 2.82, compared to 7.32, SD ± 5.94, *T*-test, *T* = 1.606, *p* = 0.118), or hours since last medication dose (3.44, SD ± 2.09, compared to 4.50, SD ± 4.86, *T*-test, *T* = 0.569, *p* = 0.574). However, there were significant differences between the distribution of self-identified gender and handedness between these two groups (Chi-square tests *p*-values: 3.56 × 10^−4^ and 0.0067, respectively).

### Collection of MDS-UPDRS Scores

For patients with PD, MDS-UPDRS-III scores were assessed immediately prior to administration of the tablet tracking task by one of two board-certified neurologists at Rhode Island Hospital with subspecialty training in movement disorders. This uniform sequence ensured that MDS-UPDRS assessments and task completion occurred in approximately the same drug state, and that task performance did not bias the assessment of the clinician. Additionally, the assessing clinician was not present during the administration of the task. MDS-UPDRS-III scores were obtained for all 26 of the patients with PD and without DBS. Same-day MDS-UPDRS-III scores were available for 24 of the 26 patients with PD who completed the task.

### Testing of Patients in “On” and “Off” DBS Stimulation States

Patients with DBS implants were alternately assigned to begin in either the “DBS On” or “DBS Off” state. Patients completed several tasks in each stimulation state with a 15-minute, task-free “washout” period after change in DBS setting. In addition to this washout period, unrelated research tasks were also performed. Cumulatively, performance of the task in each stimulation state was typically separated by approximately one hour.

### Task Administration and Data Collection

A touchscreen tablet-based motor task was developed for the iOS system (v.11.4, Apple Inc., Cupertino, California, USA) using the Swift programming language (v.4.1, Apple Inc., Cupertino, California, USA) and XCode integrated development environment (v.9.2, Apple Inc., Cupertino, California, USA). The task presented a continuously-moving target designed to capture goal-directed movement. The target path was calculated stochastically using an algorithm derived from the cubic Bezier curve equation:

$$\begin{array}{l} B\left(t\right)={\left(1-t\right)}^{3}P_{0}+{3\left(1-t\right)}^{2}tP_{1}+3{\left(1-t\right)t}^{2}P_{2}+t^{3}P_{3} \end{array}$$

where 0 ≤ *t* ≤ 1, starting point *P*_0_, endpoint *P*_3_, and two semi-random control points *P*_1_ and *P*_2_.

Twenty curves were generated and sequenced into a single continuous path by setting the endpoint of a given curve equal to the starting point of the subsequent curve. Each control point was plotted along the arc of a theoretical circle containing the previous point as the center. The radius of each control point was randomly selected from a range of 2.0–2.4 cm, and the curvature of each control point was selected from a range of 60–75°. The directionality of each curve (clockwise or counterclockwise) was determined randomly unless the target was approaching one of the screen bounds, in which case the path curved away from the edge of the screen. Furthermore, the control points were restricted to collinearity to prevent sharp “kinks” in the path.

The final path was rendered using the Swift UIBezierPath “spline” function, and the target was animated along the path at a constant speed of 4.25 cm per second. Subjects were asked to track the target (a circle with a radius of 4.0 mm) using a pressure-sensing stylus with the dominant hand. The task session was divided into 15 trials, each approximately 25 seconds in duration. The coordinates of both target and subject movements were sampled at a frequency of 100 Hz.

### Metric Calculations

Metrics were crafted to capture the heterogeneity of motor dysfunction at each time point *t*. Many of these metrics are based on previous work that employed a similar approach to motor evaluation (33), albeit in intraoperative patients undergoing DBS implantation with a task that used a joystick, rather than a stylus, to capture data. Notably, this work did not assess patients with ET, nor did it test patients in different stimulation states.

Here, data were divided into 1-second, non-overlapping epochs, and metrics were calculated for each epoch. The equations used to calculate each metric are shown in Table 2. Seven of the eight metrics were calculated using positional data, while “Pressure” reflects variance in the “force” data captured at the stylus-tablet interface. “Distance” indicates the Euclidean distance from the target trace to the cursor trace. “Tremor” corresponds to the magnitude of the 3–10 Hz tremor in the cursor trace. “VectorError” calculates the magnitude of the difference vector between the cursor and target trace vectors. “TrackingAngle” measures the angle between the cursor and target trace vectors. “Slowness” is an exponentially-transformed measure of velocity such that the maximum curvature occurs at the 80^th^ percentile of velocity. “Speed Difference” is the difference between the speed of the cursor trace and the speed of the target trace. Finally, “Excursion Difference” calculates the Euclidean distance between the cursor trace and the origin (0, 0). The non-tremor metrics were calculated using a 3 Hz low-pass filtered trace of the subject's movements to minimize the possibility of a confounding contribution of tremor.

**Table 2:** Equations used to calculate metrics used to train the SVM classifiers.

**Metric**	**Definitions**	**Equation**
Distance	–	$D\left(t\right)=\;\sqrt{{\left(x_{C}\left(t\right)-x_{T}\left(t\right)\right)}^{2}+{\left(y_{C}\left(t\right)-y_{T}\left(t\right)\right)}^{2}}$
Tremor Magnitude	${\tilde{\cdot }}_{C}{\left(t\right)}^{2}\;$is the analytic signal of the 3–10 Hz filtered cursor timeseries	$TM\left(t\right)={\tilde{x}}_{C}{\left(t\right)}^{2}+{\tilde{y}}_{C}{\left(t\right)}^{2}$
Vector Error	–	*VE*_*i*_ = |*C*_*i*_ − *T*_*i*_|
Tracking Angle	–	$TA_{i}={\text{cos}}^{-1}\left(\frac{C_{i}\;\cdot T_{i}}{\left|C_{i}||T_{i}\right|}\right)$
Slowness	*b* = −0.042	$S_{i}^{slow}=\exp\left(\frac{b\;\cdot |C_{i}|}{\Delta t_{i}}\right)$
Speed Difference	–	$S_{i}^{d}\left(t\right)=\frac{\left|C_{i}\right|}{\Delta t_{i}}-\frac{\left|T_{i}\right|}{\Delta t_{i}}$
Excursion Difference	–	*Ex*(*t*) = |*C*|
Pressure	Mean variance of the force captured by the iPad over the course of each epoch	–

**Supplementary Definitions** Let “target trace” refer to the curve traced out by the target, and let “cursor trace” refer to the curve traced out by the cursor. Given a time $t\in {\left\{t_{i}\right\}}_{i=0}^{T}$, define: *x_C_(t), y_C_(t):x and y coordinates of cursor trace*. *x_T_(t), y_T_(t):x and y coordinates of target trace*. Further, given the i^th^ time (t) bin of $\Delta t\in {\left\{\Delta t_{i}\right\}}_{i=1}^{T}$, define: *C_i_* ≜ *(x_c_ (i) − x_C_(i − 1), y_C_ (i) − y_C_ (i − 1)) : vector representing the cursor trace for time bin, i*. *T_i_* ≜ *(x_T_ (i) − x_T_ (i − 1), y_T_ (i) − y_T_ (i − 1)) **:** vector representing the target trace for time bin, i*.

### Support Vector Machine Analysis

For *n* metrics, the epochs of a subject trace were transformed into a vector of metrics, ${{\text{m}}_{\text{i}}\in R}^{n}$, where *R* is the set of real numbers, to the formulas in Table 2. To classify the points of a subject's trace as symptomatic or asymptomatic, an SVM was trained for each subject in *R*^*n*^. The points of the control traces were labeled “non-movement disorder-associated,” while the points of a movement disorder patient trace were labeled “movement disorder-associated.”

Given the large size of the pooled control subject dataset compared to the single patient dataset to which it was compared, a Monte Carlo method was employed to reduce control bias. For each iteration of this method, control points were randomly subsampled (without replacement) by a factor of $\frac{1}{20}$ to yield a 1:1 ratio of symptomatic to non-symptomatic points (this denominator reflects the total number of control subjects). For each classifier, an SVM with a linear kernel was fit to 80 percent of the data with 10-fold cross-validation as a “training” dataset, to generate a hyperplane in *R*^*n*^ with coefficients $h_{n}^{i}\in R^{n+1}$ for iteration *i*, with a constant 0^th^ coordinate, and the 1^st^ through *n*^th^ coefficients corresponding to the coefficient of each metric. This process was repeated 100 times, and average of the coefficients ***h***^*i*^ were used to produce a hyperplane with coefficients $h=\;\frac{\sum_{i=1}^{100}h^{i}}{100}$. The SVMs were fit using scikit-learn 0.19.1 (34). The remaining 20 percent of the data were used as a test set for the classifier; validation accuracies are reported from this test dataset. However, “motor error scores” (MES) were calculated from a classifier trained on all of the data to maximize the yield of our dataset. SVM hyperparameters were selected in advance of any of these analysis and were not tuned to individual patients to minimize the possibility of overfitting.

The degree of motor dysfunction at a point was measured by the signed Euclidean distance from that point to the hyperplane; we called these values MES. A positive MES corresponded to increased motor dysfunction. That is, given the coefficients ***h*** of a hyperplane and a subject vector ***m***_***i***_,

(1) $$\begin{array}{l} SS_{i}=\;\frac{h_{1:\text{n}}\cdot {\text{m}}_{\text{i}}+h_{0}}{\left|h_{1:\text{n}}\right|} \end{array}$$

where ***h***_**1:n**_ is a vector with the 1st to *n*th coordinates of ***h***, and ***h***_**0**_ is a constant with the first coordinate of ***h***.

The weight of a metric was defined as the square of its corresponding coefficient divided by the sum of the squares of the coefficients. Thus, the weight of the *i*^th^ metric is given by:

(2) $$\begin{array}{l} w_{i}=\frac{{h_{i}}^{2}}{\sum_{j=1}^{1}{h_{j}}^{2}}. \end{array}$$

### Other Analyses and Plot Generation

All other statistical analyses were performed using the “stats” library from SciPy (35), and all graphs were generated using the Matplotlib (36) and seaborn libraries (www.seaborn.pydata.org). All analysis and plotting scripts were executed using Python 3 (www.python.org).

### Data and Code Availability

De-identified data and analysis code are available upon request for use in collaboration.

## Results

### Non-redundant Metrics Were Used to Calculate an Inclusive “Motor Error Score”

**Figure 1:**
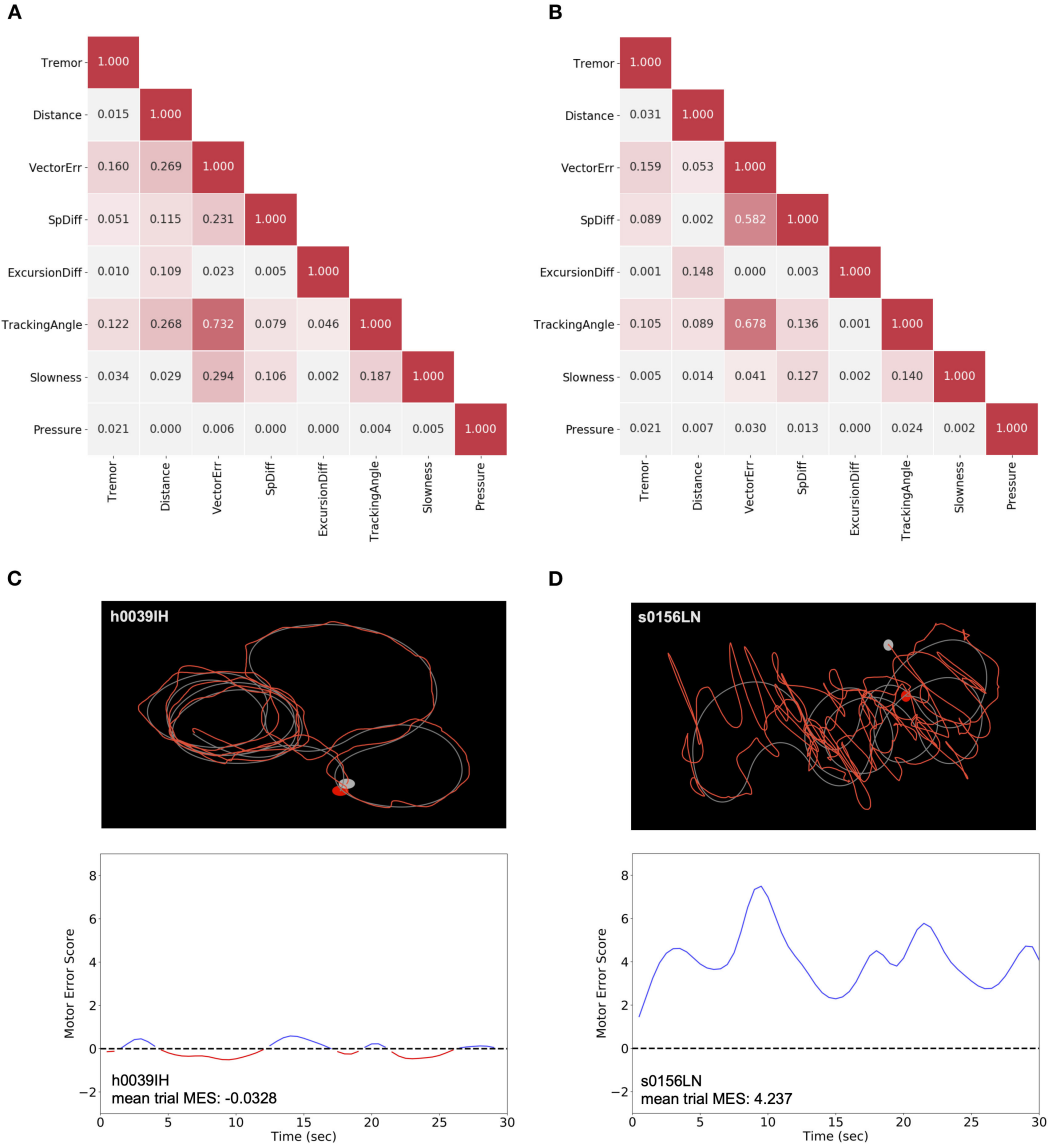
Generation of MES using non-redundant metrics. **(A,B)** For each subject, the distribution for each metric was normalized to the pooled control subject data for that metric. The strength of the association was compared between each pair of normalized metric means across all control subjects **(A)** and patients with PD **(B)**. Pearson's *r*^2^ are shown to indicate the strength of correlation. **(C,D)** The raw trace data from individual trials (top) of control subjects like h0039IH **(C)** and patients with PD, like s0156LN **(D)** were used to calculate the eight metrics. These metrics were then used to generate an SVM classifier model by comparing a single subject to a sampling of pooled control subject data. The distance from each point of patient data to the hyperplane for a given epoch corresponds to the MES, which serves as an aggregate, scalar measure of motor dysfunction across a representative trial (bottom). Here, a Gaussian smoothing function was applied to the MES for visualization purposes.

Using raw positional and pressure data epochs collected during each trial, eight metrics were calculated (Table 1). Across the entire group of either control subjects (Figure 1A) or patients with PD (Figure 1B), correlations between metrics were calculated to assess for potential redundancy. For each metric pair, Pearson's *r*^2^ was calculated. In both of these groups, “Tracking Angle” and “Tremor” showed *r*^2^ values > 0.7; however, no other metric pairing showed a strong correlation. The overall independence of the metrics suggests that each captures a different component of motor dysfunction, and correlation analysis of the relationships between metrics and MDS-UPDRS sub-scores revealed some evidence, albeit not statistically significant in this sample, in support of this possibility (Supplementary Figure 1).

Using these eight metrics, we used SVM, a linear machine learning algorithm, to generate a classifier to differentiate patients with PD and control subjects. Specifically, we produced classifiers to discriminate individual movement disorder subjects from the pooled performance data across control subjects. From these models, we generated a set of “Motor Error Scores” (MES) for each patient, which corresponded to the distance between a given patient's data points and the SVM hyperplane. Thus, these MES are scalar measures of motor dysfunction that capture the constellation of movement abnormalities to reflect the cumulative severity of a patient's disease manifestation in short epochs (Figures 1C,D). To assess the true effectiveness of the SVM-generated classifier, we re-ran the classification on our cohort of clinic patients with PD or ET after randomly shuffling the labels applied to each subject (“control” or “patient”). This label-shuffling resulted in highly significant decreases in classification accuracy, from 0.839 (SD ± 0.140) to 0.477 (SD ± 0.0828) in the PD-control comparison and from 0.892 (SD ± 0.187) to 0.506 (SD ± 0.113) in the ET-control comparison. This loss of specificity with label shuffling indicates that our task and analytic approach differentiated specifically between control subjects and patients with motor dysfunction (Supplementary Figure 2, Wilcoxon signed-rank tests, *W* = 0.0 and *p* = 8.256 × 10^−6^, *W* = 0.0 and *p* = 0.00221 for PD and ET analyses, respectively).

### Multi-dimensional Metric-Based Analysis Correlated With Clinician-Assessed MDS-UPDRS-III Scores

**Figure 2:**
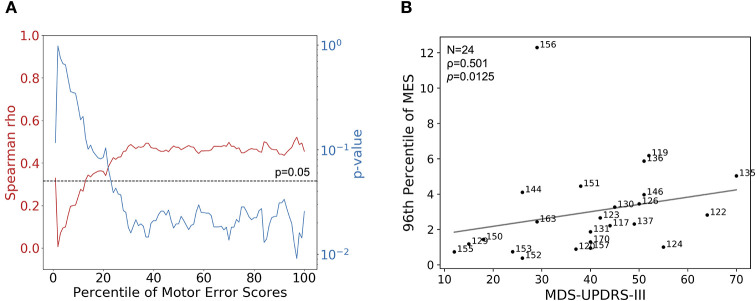
Correlations of MES with MDS-UPDRS-III. **(A)** Using MDS-UPDRS-III, Spearman's correlation coefficient (red line) and corresponding *p*-values (blue line) were calculated using percentiles between 0 and 100 of session-wide MES for each patient. Higher percentiles correspond to progressively smaller fractions of higher-valued MES. The dotted line indicates a *p*-value of 0.05, the selected alpha-level in this analysis. In general, better correlations between MES and MDS-UPDRS were observed when considering patients' epochs of more prominent motor dysfunction; the maximum correlation and lowest *p*-value occurred at the 96^th^ percentile of MES. **(B)** MDS-UPDRS-III (Motor Examination subsection) scores plotted with corresponding 96^th^ percentile session-wide MES for 24 patients with PD for which MDS-UPDRS-III scores were available. Strength of association was determined by calculating Spearman's correlation coefficient (ρ = 0.501, *p* = 0.0125).

Of the 26 patients with PD who performed the behavioral task, same-day clinical MDS-UPDRS-III assessments were available for 24. In all cases, patients underwent the clinical assessment and completed the behavioral task in the same medication state, as described in **Materials and Methods**. We assessed the correlation between MDS-UPDRS-III score and a broad range of percentiles of SVM-generated MES. We calculated the MES for each percentile between 1 and 100 for each patient (a patient's median MES would be represented by the 50^th^ percentile). Then, for a given percentile, the MES for each patient were correlated with their MDS-UPDRS-III scores, and Spearman's rank order correlation analysis was performed. From this analysis, we observed that the *p*-value for this analysis dropped below the pre-selected alpha level of 0.05 near the 20^th^ percentile of MES. The *p*-value of this analysis reached its minimum (*p* = 0.0125) at the 96^th^ percentile of MES. Around this same percentile, we also observed the maximum correlation coefficient (ρ = 0.501) (Figures 2A,B). This analysis suggests that clinicians were likely generating their clinical assessments based more closely on their perception of a patient's maximum symptom severity. There was no correlation between the MES and the patients' point in their inter-dose interval at the time of task completion (Spearman ρ = −0.0969, *p* = 0.676).

A similar analysis was performed using the sum of all components of the MDS-UPDRS-III that assessed symptom severity in the dominant upper extremity (DUE), given that the tracking task collects data related to symptoms only affecting this extremity. In this case, the maximum Spearman's ρ and the minimum *p*-value occured at the 98^th^ percentile of MES, and were 0.351 and 0.0929, respectively (Supplementary Figure 3B).

### Distributions of MES Were Effective Differentiators Between Subject Types

**Figure 3:**
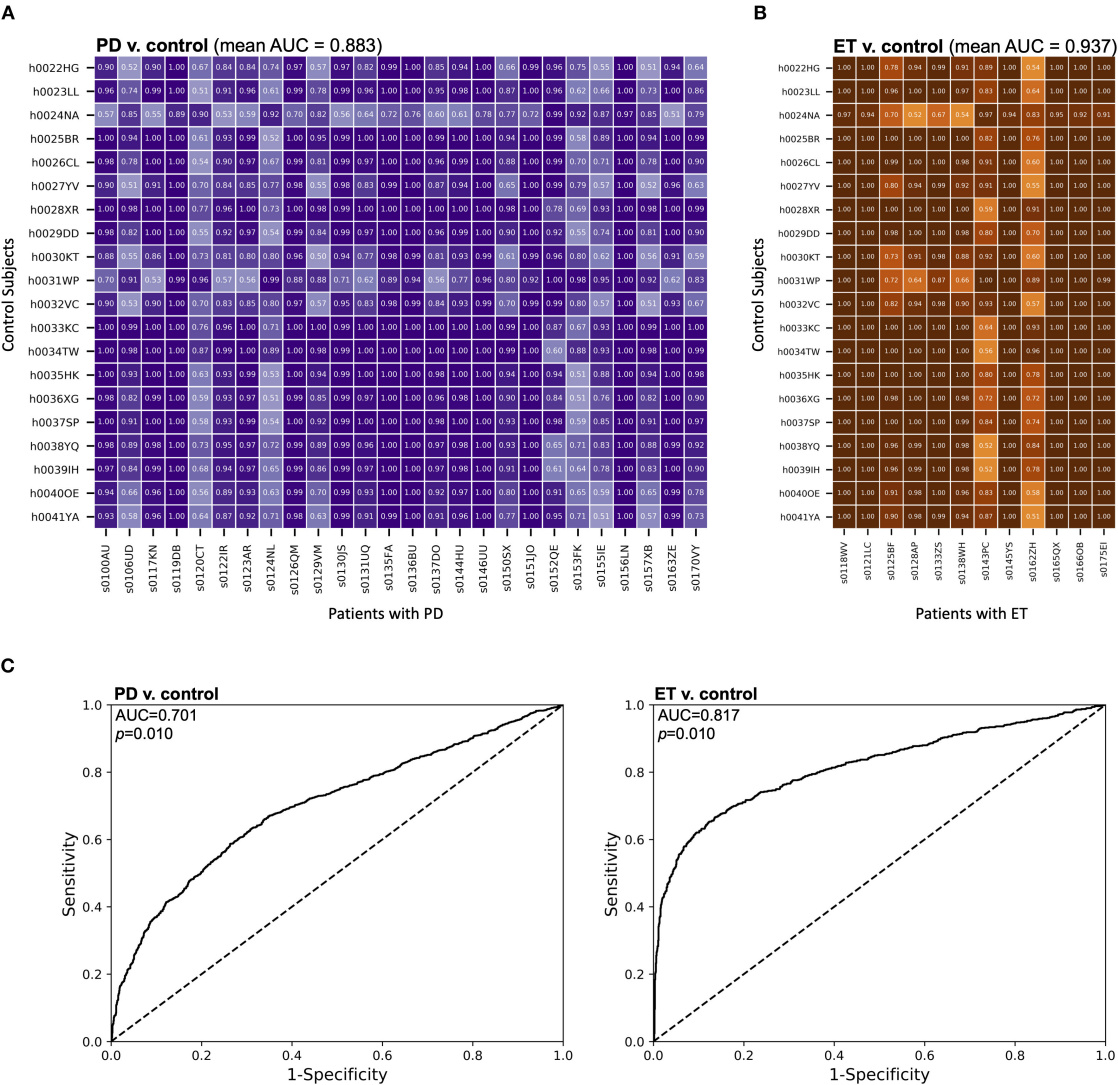
MES effectively discriminated between movement disorder patients and control subjects. **(A,B)** The distributions of MES over a session were compared between individual control subjects and **(A)** patients with PD or **(B)** patients with ET. Here, unique SVM models were generated for each comparison and the resulting MES for each control subject-patient combination were calculated based on these models. These MES distributions were then compared using a ROC analysis. The AUC for each comparison was then calculated. AUCs for each pair-wise comparison are shown. **(C)** ROC curves were generated from compiled MES distributions from each subject group. All MES were derived from an SVM comparison of individual subjects within a group with a random sampling of pooled control data. The AUCs were then calculated to quantify the discriminatory ability of each comparison.

To assess the ability of MES to classify individual epochs as symptomatic or asymptomatic, we performed pairwise analyses between each individual patient and control. For each pair, a unique classifier was generated using SVM as described above. The MES distributions generated from these SVMs were analyzed using receiver operating characteristic (ROC) curves, and discrimination between the distributions was quantified using the area under these ROC curves (AUC). When comparing patients with PD to control subjects using this method, the mean AUC across all pairs was 0.883 (SD ± 0.149), indicating good discrimination between these two subject types at the 1-second epoch timescale (Figure 3A). Lower AUCs were generally grouped by patient with PD, suggesting that these particular patients had less motor dysfunction at the time of task performance. Importantly, there are no similar groupings by control subject, suggesting lower variability of performance across this group. A similar comparison of patients with ET to control subjects yielded an even higher mean AUC of 0.937 (SD ± 0.122) (Figure 3B), meaning that, for these cohorts, the task differentiated patients with ET from control subjects significantly better than it did for patients with PD (Mann-Whitney test, *U* = 37,899 and *p* < 0.0001). Like the analysis of patients with PD, lower AUCs were generally grouped by patient and not by control subject.

We also compared MES distributions of different groups of subjects at the population level (Figure 3C). All MES were derived from SVM classification compared to a random sampling of the pooled control subject data. We then calculated the area under these curves (AUC) to assess the discriminability of experimental groups based upon MES. In our comparison of the MES distributions of patients with PD and control subjects, we found an AUC of 0.701 (*p* = 0.010 by bootstrapping with 100 re-samplings). A similar comparison of patients with ET and control subjects yielded an AUC of 0.817 (*p* = 0.010 by bootstrapping with 100 re-samplings). Therefore, in addition to discriminating between epochs in individual patients, our approach has the ability to discriminate between patients with movement disorders and control subjects on a population level.

### Metric-based Analysis Highlighted Motor Differences Between PD and ET Patients

The SVM algorithm returns a “weight” for each metric that reflects its relative contribution to generating the classification hyperplane. Thus, these “metric weights” should approximate the heterogeneity of the motor dysfunction in each movement disorder patient. SVM comparison of individual patients with PD and pooled control subjects showed a high degree of such heterogeneity. This diversity was reflected in the relative variability in metric weights in patients with PD and patients with ET. Mixed effect analysis of these data showed a significant interaction between the metrics and the different movement disorders (*F* = 9.39, *p* < 0.0001). Multiple *T*-tests comparing metric weights of patients with PD and patients with ET with *p-*values adjusted using the Bonferroni-Dunn method showed significant differences between “Tremor” and “Distance” at the *p* < 0.05 level (*t* ratios were 3.947 and 3.771; adjusted *p-*values were 0.00281 and 0.00467, respectively) (Table 3).

**Table 3:** Comparison of metric weights between patients with PD and patients with ET.

**Metric**	***t* ratio**	**Adjusted *p*-value**
**Tremor**	**3.947**	**0.00281**
**Distance**	**3.771**	**0.00467**
VectorErr	2.317	0.211
SpDiff	1.645	0.869
ExDiff	1.561	1.00
TrAngle	1.569	1.00
Slowness	0.414	1.00
Pressure	0.107	1.00

*Multiple t-test results with correction using the Bonferroni-Dunn method comparing metric weights between patients with PD or ET, each compared to pooled control subject data. Two-tailed p-values are given. Significant results in bold*.

**Figure 4:**
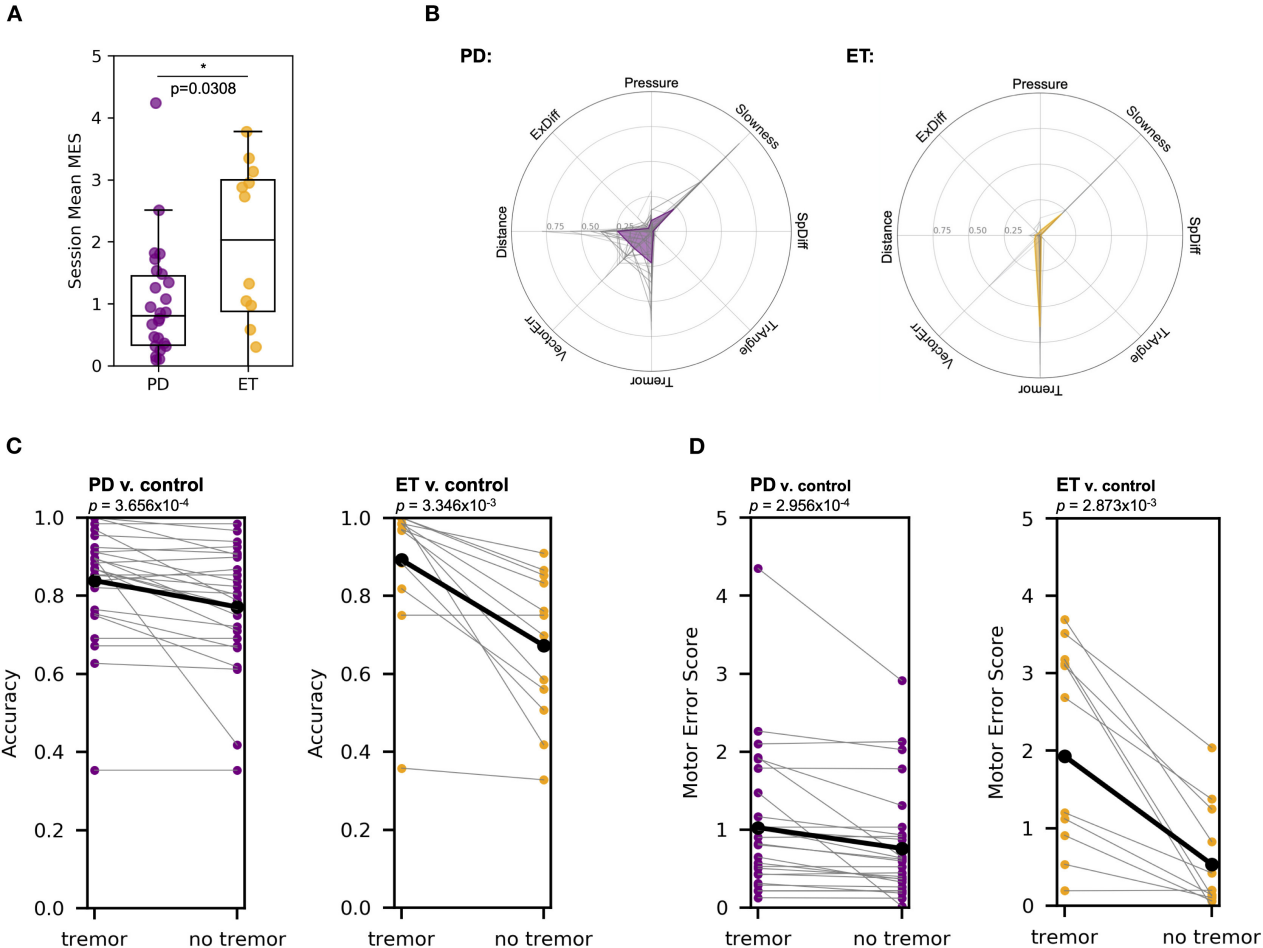
MES metric weights, particularly tremor, differed between patients that have PD compared to ET. **(A)** Mann-Whitney U test showed that patients with ET have a significantly higher mean MES on a session-to-session basis than patients with PD (U = 96.0, *p* = 0.0308). Each point represents the mean MES across a single session for a given patient. **(B)** For the SVM classifier developed for each patient compared to pooled control subject data, the relative contribution of each of the seven different metrics varied. The left plot shows the metric weights calculated for each patient with PD (gray lines) and the mean metric weights across all patients with PD (purple line). The right plot shows a similar analysis for patients with ET (orange line). **(C)** Removing the “Tremor” metric from the SVM algorithm reduced the accuracy of the resulting classifier in PD v. pooled control (left, purple) and ET v. pooled control (right, orange) comparisons (Wilcoxon signed-rank tests, test statistics were 13.0 and 0.0, while *p*-values were 3.656 × 10^−4^ and 3.346 × 10^−3^, respectively). **(D)** Removing the “Tremor” metric from the SVM algorithm reduced the MES generated in PD v. pooled control (left, purple) and ET v. pooled control (right, orange) comparisons (Wilcoxon signed-rank tests, test statistics were 28.0 and 1.0, while *p*-values were 2.956 × 10^−4^ and 2.873 × 10^−3^, respectively).

On average, patients with ET demonstrated higher MES than those with PD (1.931, SD ± 1.407 compared to 1.027, SD ± 0.945, respectively; Mann-Whitney U test, U = 96.0, *p* = 0.0308) (Figure 4A). We hypothesized that the unique constellation of motor abnormalities observed in PD and ET might contribute to the differentiation between these patients in our analysis. Specifically, the differentiation of patients with ET from control subjects relied heavily on the “Tremor” metric (Figure 4B), while the metric most important for differentiation between patients with PD and control subjects varied in individual cases. To further interrogate these differences, we compared each patient with PD to a random sampling of pooled data from patients with ET. We chose to use the patient with ET data analogously to the control data in this analysis because the ET-control SVM analysis showed relatively homogenous metric weights relative to patients with PD, suggesting less motor feature variability within this cohort. In this analysis, the SVM distinguished individual patients with PD from the pooled ET subject data with an accuracy of 0.924 (SD ± 0.0764), consistent with the ability of our multi-dimensional approach to motor analysis to differentiate between these movement disorders despite some possible overlap in clinical presentation, specifically with regard to tremor.

To confirm the relative importance of tremor in classifying patients with ET compared to those with PD, we repeated our SVM analyses without including the “Tremor” metric (Figure 4C). In the PD-control SVM analysis, excluding “Tremor” reduced the classification accuracy from 0.839 (SD ± 0.140) to 0.772 (SD ± 0.155) (Wilcoxon signed-rank test, *W* = 13 and *p* = 3.656 × 10^−4^), while a “Tremor”-excluded ET-control SVM analysis reduced mean classification accuracy from 0.892 (SD ± 0.187) to 0.672 (SD ± 0.190) (Wilcoxon signed-rank test, *W* = 0.0 and *p* = 3.346 × 10^−3^). Finally, removing the “Tremor” from the PD-pooled ET SVM comparison reduced classification accuracy from 0.924 (SD ± 0.0764) to 0.760 (SD ± 0.140) (Wilcoxon signed-rank test, *W* = 0.0 and *p* = 8.277 × 10^−6^). The mean accuracy difference (with vs. without the “Tremor” metric) in patients with PD was significantly less than the mean accuracy difference in patients with ET (*T*-test with Welch's correction, *T* = 2.872 and *p* = 0.0117). These analyses indicate that “Tremor” is a more critical metric for differentiation of patients with ET from pooled control subject data compared to classification of patients with PD, consistent with the expected phenotypic dominance of the tremor in patients with ET and the more heterogenous clinical phenotype of patients with PD.

We then analyzed the changes in MES after the removal of “Tremor” from the SVM algorithm (Figure 4D). In the PD-control analysis, the mean MES for patients with PD decreased from 1.027 (SD ± 0.945) to 0.760 (SD ± 0.731), a statistically significant change (Wilcoxon signed-rank test, *W* = 28.0 and *p* = 2.956 × 10^−4^). In the ET-control analysis, the mean MES for patients with ET decreased from 1.931 (SD ± 1.407) to 0.535 (SD ± 0.684), also a significant change (Wilcoxon signed-rank test, *W* = 1.0 and *p* = 2.873 × 10^−3^). The mean MES difference was significantly lower in patients with PD compared to those with ET (*T*-test with Welch's correction, *T* = 3.382 and *p* = 0.0051), again consistent with the notion that tremor accounts for a larger component of overall motor dysfunction in ET than in PD.

Examination of these data on the level of individual patients further illuminates the relative differences in the diversity of motor manifestations between patients with PD and ET. In both the MES and classification accuracy analyses, all but two of the 12 patients with ET showed a sharp decline in the “Tremor”-excluded analysis, and the two patients that did not show this decline had mild baseline motor impairment according to their tremor-inclusive MES (0.0562, SD ± 0.195). This observation confirms that, in patients with ET who are relatively symptomatic, tremor is the dominant clinical phenotype.

### Multi-dimensional SVM Classification Differentiated Between DBS States

We applied our tablet-based task and multi-dimensional SVM analysis to nine patients with PD with implanted DBS systems to determine the task's ability to differentiate between DBS states within individual patients. One of these nine patients was excluded due to their inability to complete the task in the “DBS Off” state due to symptom severity. Characteristics of the eight patients who completed the task in both stimulation states are described in Table 2.

**Figure 5:**
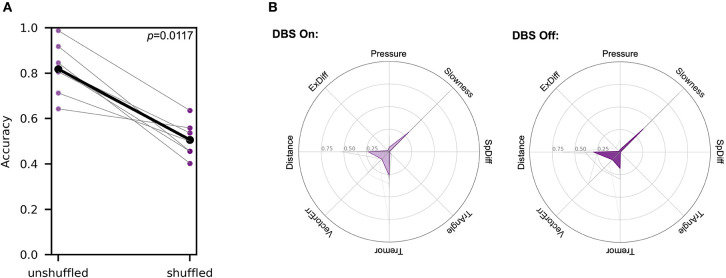
SVM analysis differentiated between DBS states in symptomatic patients. Each patient was tested in both the “DBS On” and “DBS Off” states within the span of 1 h. The order of DBS states tested alternated between patients. **(A)** Accuracies of a single SVM classifier comparing individual patients in two different stimulation states are compared to accuracies produced using an analogous SVM analysis, but with random label shuffling prior to hyperplane generation (Wilcoxon signed rank test, *W* = 0.0 and *p* = 0.0117). **(B)** Metric weights generated by PD patient-pooled control data SVM comparisons were used to examine the relative contributions of each metric in the two different DBS states. The left polar plot depicts the mean metric weights of patients in the “DBS On” state. and the right plot shows the weights of patients in the “DBS Off” state. In each plot, gray lines represent metric weights for individual patients. A two-way ANOVA analysis of these data showed no significant interaction between the metrics and the different movement disorders (*F* = 1.966, *p* = 0.0762).

Patients were alternately assigned to perform the task first either in the “DBS On” or “DBS Off” state to reduce the impact of learning with task repetition affecting results. A single SVM classifier was generated to distinguish between stimulation states within a single patient. The accuracies generated from this analysis were compared to the accuracies produced from an SVM-based analysis for each patient in the DBS state in which labels prior were shuffled prior to generation of the hyperplane. A pair-wise comparison of these analyses showed that shuffling of labels significantly decreased classification accuracy from 0.819 (SD ± 0.108) to 0.507 (SD ± 0.0720) (Wilcoxon signed rank test, *W* = 0.0 and one-tailed *p* = 0.0117) (Figure 5A), indicating that the SVM effectively distinguished between the two stimulation states.

Separately, we generated classifiers to differentiate between each patient in each stimulation state and pooled control subject data to analyze the differences in metric weights. A two-way ANOVA analysis of these data showed no significant interaction between the metrics and the different movement disorders (*F* = 1.966, *p* = 0.0762) (Figure 5B).

## Discussion

Using data captured from our naturalistic, goal-directed task and an eight-dimensional, metric-based analysis of these data, we found that, across a cohort of patients with PD, our multi-dimensional “MES” correlated with the cumulative score of the Motor Examination subsection of the MDS-UPDRS. This indicates that our method of motor dysfunction assessment can approximate the severity of a patient's condition when compared to the standardized assessments of trained clinicians. While other tools developed for quantification of motor dysfunction correlate more strongly with MDS-UPDRS-III (37), we did not specifically design our task to optimize this relationship. Rather, our goal was to achieve maximal differentiation between normal and abnormal goal-directed movement. Any correlation with MDS-UPDRS-III, in other words, was incidental. While we did not observe correlations between specific metrics and components of the MDS-UPDRS-III, increased data collection across centers facilitated by the objectivity and usability of our task may reveal a relationship between our metrics and MDS-UPDRS-III subscore “factors” of motor dysfunction (11).

Our task's correlation with MDS-UPDRS-III lends it validity, but it was specifically designed to assess motor dysfunction with high temporal precision and thus provide insight into the short-timescale fluctuations in PD symptomatology (38–40). Importantly, such high-resolution temporal measurement of these fluctuations in motor dysfunction is necessary to correlate behavioral phenotypes with neural activity, a crucial step in the development of adaptive or closed-loop DBS systems (41–44). Additionally, this task can be performed in a non-clinical setting, meaning that the frequency of data collection need not be limited to the interval between office visits. More frequent assessment of symptomatology might allow for more robust projection of trends and more timely implementation of beneficial therapeutic changes (16, 18, 24–26).

Analysis of MES distributions offered strong evidence that the task differentiated between control subjects and movement disorder patients using 1-second epochs of motor performance. However, it is important that such a tool detects motor dysfunction specific to the movement disorder of interest. A direct comparison of patients with PD or ET within a single SVM model showed that differences in metric weight patterns across these groups can be used to generate a high-accuracy classifier. Using linear SVM models allowed us to examine the relative contributions of several different measures of performance to the overall MES. In comparing patients with PD to those with ET, these metric weights reflected broad clinical distinctions between the diseases. Within individual patients and across the entire group, patients with PD showed greater symptom heterogeneity, while MES generated for patients with ET relied predominantly on the “Tremor” metric. Comparison of metric weights across groups confirmed that “Tremor” differs the most among subject groups, although “Distance” also differed significantly (Table 3).

Thus, our task and corresponding panel of metrics not only distinguished patients with movement disorders from those without movement disorders, but that it is capable of discriminating between movement disorders directly. Although the textbook clinical pictures of these two movement disorders are distinct, misdiagnosis persists in both directions (45). Some quantitative diagnostic tools designed to distinguish between PD and ET are accelerometer-based and rely on differences in tremor characteristics alone, such as frequency (19, 46, 47). Others require significant training for proper administration, such as those that use electromyography or transcranial sonography (48, 49). Although our task specifically targets PD-associated motor dysfunction without the goal of *de novo* diagnosis, it is an easily-implemented test that can discriminate between ET and PD, even in cases where tremor characteristics may be ambiguous (50, 51).

Importantly, generating classifiers for individual patients proved effective at differentiating between stimulation states within individual patients with PD. Ultimately, the purpose of a temporally precise, highly quantitative symptom assessment is to correlate behavior with neural activity in order to guide adaptive neuromodulation therapy. This task's ability to detect behavioral changes that correspond to different stimulation states suggests that it has potential to effectively contribute to the understanding of the neural changes related to motor dysfunction in movement disorders.

This study had several limitations. On average, patients with PD were significantly older and more male than control subjects, highlighting two potential confounding factors. Additionally, the task only assesses symptomatology in the dominant upper extremity, which introduces two possible sources of error. Firstly, PD motor dysfunction is generally unilateral, particularly in early stages of the disease, while the tremor of ET is generally bilateral. In our assessment, it is feasible that a patient with PD may have more severe motor dysfunction in their non-dominant hand, while we capture the milder dysfunction in their dominant hand. Secondly, as compared to the MDS-UPDRS-III, our task does not provide a global assessment of the patient's motor dysfunction. We do not assess lower limbs, face, speech, or several other components of this scale. Also, despite screening for significant cognitive impairment, we cannot exclude the possibility that cognitive dysfunction, a well-known sequela of late-stage PD, may affect task performance in a way that our analysis may mistake for pure motor dysfunction. Finally, the tremor phenotypes characteristic of various movement disorders may be unequally assessed by this task. For example, resting tremor is considered a hallmark symptom of PD, although action tremor is still a common finding (52) that correlates with the severity of resting tremor and rigidity, suggesting that it is more likely to be present in advanced disease. Given the small magnitude of movements captured by our task, it likely assesses components of both action and postural tremor. Thus, it may be less effective in patients in earlier stages of PD without components of these two tremor features. Additionally, the action tremor captured through a goal-directed task aligns more closely with the typical clinical phenotype of patients with ET, highlighting the potential problems of a direct comparison of MES of patients with PD and ET. Our study is also limited in terms of its sample sizes, particularly in the assessments of patients with PD and DBS (*N* = 8) and patients with ET (*N* = 12). By performing the task with more patients, we could further elucidate the relationship between our metrics and specific clinical symptoms of different movement disorders.

Overall, despite these limitations, our results suggest that an objective, continuous, naturalistic motor task can capture motor impairment patterns that are specific to PD and ET, can distinguish between DBS states in patients with PD, and can be used to quantify the degree of motor dysfunction with high temporal precision.

## Data Availability Statement

The raw data supporting the conclusions of this article will be made available by the authors, without undue reservation.

## Ethics Statement

The studies involving human participants were reviewed and approved by Lifespan IRB - Rhode Island Hospital. The patients/participants provided their written informed consent to participate in this study.

## Author Contributions

JS, JY, DL, DA, PL, SL, and WA designed the task and analyses. JS, JY, DA, PL, and SL administered the task. AD'A and UA performed MDS-UPDRS-III evaluations. JS and WA wrote the manuscript. All authors contributed to the article and approved the submitted version.

## Conflict of Interest

The authors declare that the research was conducted in the absence of any commercial or financial relationships that could be construed as a potential conflict of interest.

## Supplementary Material

Supplementary Figure 1 MDS-UPDRS-III sub-scores weakly correlated with motor dysfunction metrics. Raw metric data for individual patients with PD were normalized to the aggregated data of this group. Metrics were then compared to each of the shown MDS-UPDRS-III sub-scores, and Pearson's correlation coefficients were calculated. Displayed are Pearson's correlation coefficients (*r*). ^*^ indicates *p* < 0.05.

Supplementary Figure 2 SVM classifiers specifically differentiated patients with motor dysfunction from control subjects. To confirm that the SVM classification was indicative of the task's ability to broadly distinguish between patients with movement disorders and control subjects instead of simply differentiating between individuals based upon idiosyncratic task performance, PD (left) or ET (right) patient and control subject data labels were randomly shuffled before generation of the hyperplane. This shuffling decreased the mean classification accuracy from 0.839 (SD ± 0.140) to 0.477 (SD ± 0.0828) in the PD-control comparison and from 0.892 (SD ± 0.187) to 0.506 (SD ± 0.113) in the ET-control comparison (Wilcoxon signed-rank test, *W* = 0.0 and *p* = 8.256 × 10^−6^ and *W* = 0.0 and *p* = 0.00221). Each gray line represents a single patient and black lines represent means.

Supplementary Figure 3 Pooled MDS-UPDRS-III scores for the dominant upper extremity also correlate to calculated MES. **(A)** The sum of all MDS-UPDRS-III score components from each patient's dominant upper extremity (DUE) were plotted with corresponding, session-wide MES means for 24 patients with PD. Spearman's correlation coefficient (red line) and corresponding *p*-values (blue line) and were calculated using percentiles between 0 and 100 of session-wide MES per patient. Dotted line indicates a *p*-value of 0.05, the selected alpha-level in this analysis. The maximum strength of correlation and statistical significance occurred at the 98^th^ percentile of MES. **(B)** Using MDS-UPDRS-III DUE, Spearman's correlation analysis demonstrated significant association between MDS-UPDRS-III DUE and the 98^th^ percentile of session wide MES for each patient (ρ = 0.351, *p* = 0.0929).

Supplementary Table 1 Stimulation parameters of patients with PD with DBS. Electrodes were targeted to stimulate either the subthalamic nucleus (STN) or the globus pallidus internus (GPi).

## Abbreviations

ADL: activity of daily living
AUC: area under the curve
DBS: deep brain stimulation
ET: essential tremor
GPi: globus pallidus internus
MES: motor error score
PIGD: postural instability/gait difficulty
PD: Parkinson's disease
ROC: receiver operating characteristic
STN: subthalamic nucleus
SVM: support vector machine
TD: tremor-dominant
MDS-UPDRS: Movement Disorder Society-Sponsored Revision of the Unified Parkinson's Disease Rating Scale.
