# Supplementary figures and images for: Multi-Dimensional, Short-Timescale Quantification of Parkinson's Disease and Essential Tremor Motor Dysfunction
Source: Front Neurol. 2020 Sep 18;11:886. doi: 10.3389/fneur.2020.00886 (PMC7530842; doi:10.3389/fneur.2020.00886)

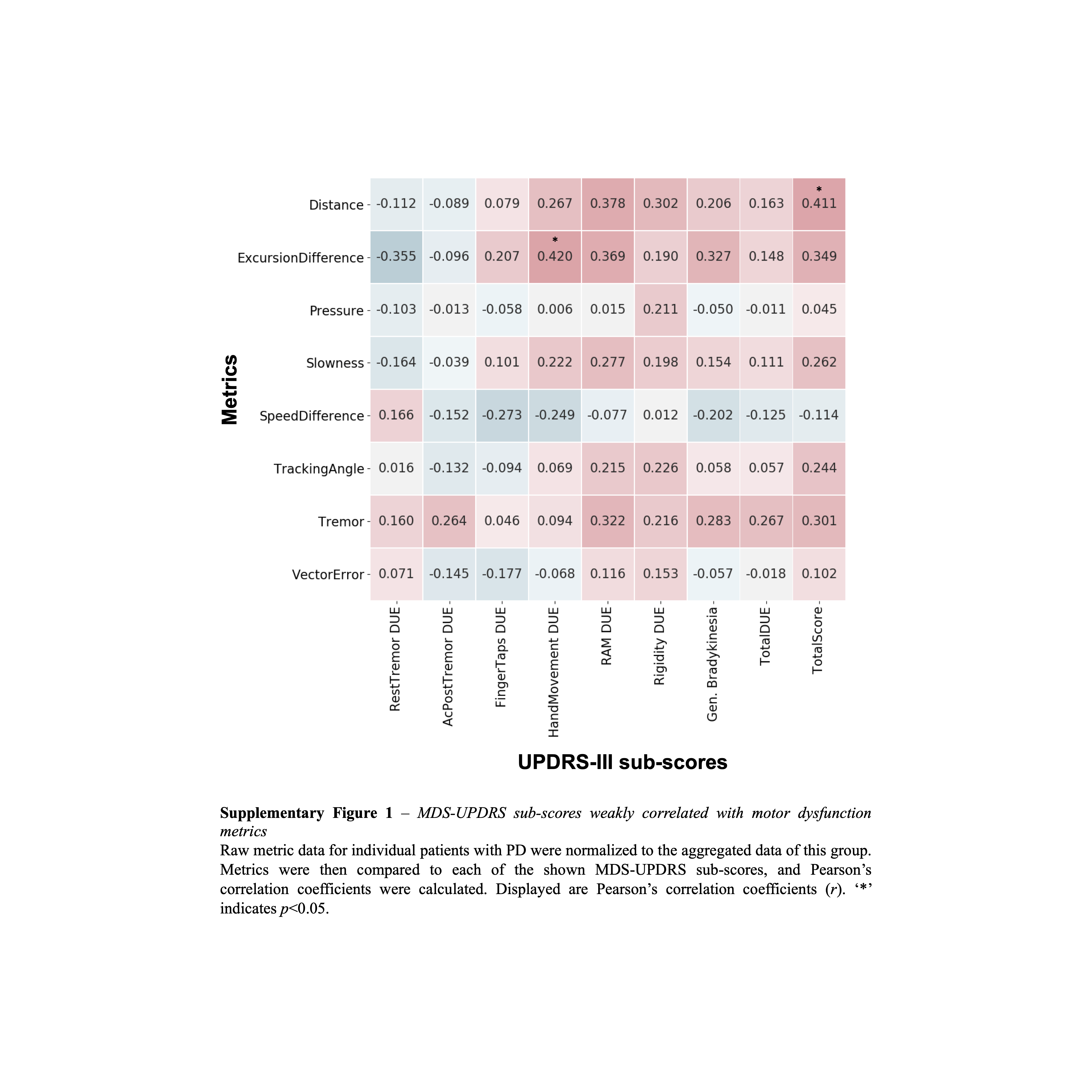

Supplement: Supplementary Figure 1 — MDS-UPDRS-III sub-scores weakly correlated with motor dysfunction metrics. Raw metric data for individual patients with PD were normalized to the aggregated data of this group. Metrics were then compared to each of the shown MDS-UPDRS-III sub-scores, and Pearson's correlation coefficients were calculated. Displayed are Pearson's correlation coefficients (r). * indicates p < 0.05. [file Image_1.tiff]

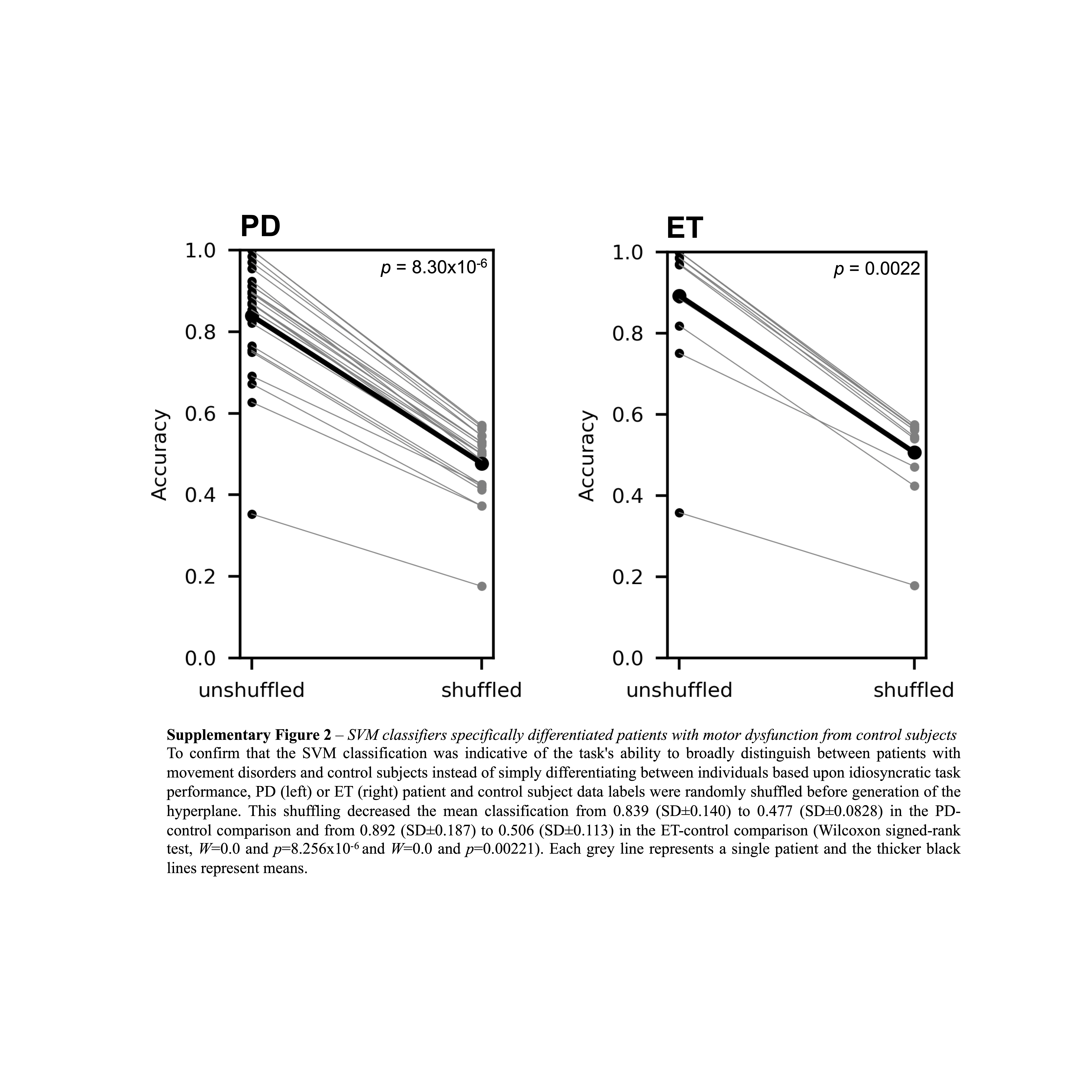

Supplement: Supplementary Figure 2 — SVM classifiers specifically differentiated patients with motor dysfunction from control subjects. To confirm that the SVM classification was indicative of the task's ability to broadly distinguish between patients with movement disorders and control subjects instead of simply differentiating between individuals based upon idiosyncratic task performance, PD (left) or ET (right) patient and control subject data labels were randomly shuffled before generation of the hyperplane. This shuffling decreased the mean classification accuracy from 0.839 (SD ± 0.140) to 0.477 (SD ± 0.0828) in the PD-control comparison and from 0.892 (SD ± 0.187) to 0.506 (SD ± 0.113) in the ET-control comparison (Wilcoxon signed-rank test, W = 0.0 and p = 8.256 × 10^−6^ and W = 0.0 and p = 0.00221). Each gray line represents a single patient and black lines represent means. [file Image_2.tiff]

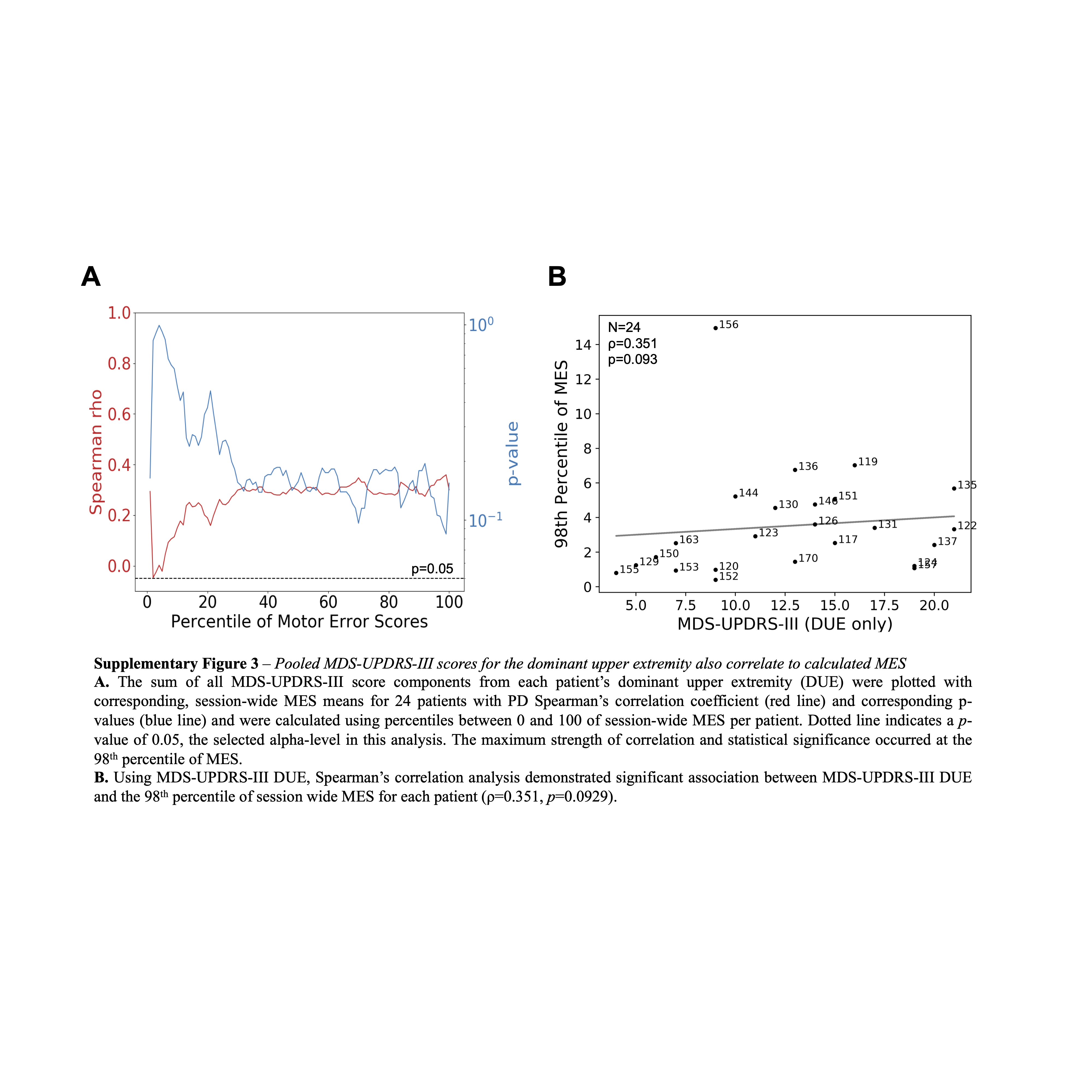

Supplement: Supplementary Figure 3 — Pooled MDS-UPDRS-III scores for the dominant upper extremity also correlate to calculated MES. (A) The sum of all MDS-UPDRS-III score components from each patient's dominant upper extremity (DUE) were plotted with corresponding, session-wide MES means for 24 patients with PD. Spearman's correlation coefficient (red line) and corresponding p-values (blue line) and were calculated using percentiles between 0 and 100 of session-wide MES per patient. Dotted line indicates a p-value of 0.05, the selected alpha-level in this analysis. The maximum strength of correlation and statistical significance occurred at the 98th percentile of MES. (B) Using MDS-UPDRS-III DUE, Spearman's correlation analysis demonstrated significant association between MDS-UPDRS-III DUE and the 98th percentile of session wide MES for each patient (ρ = 0.351, p = 0.0929). [file Image_3.tiff]
