# Supplementary material for: Multi-Dimensional, Short-Timescale Quantification of Parkinson's Disease and Essential Tremor Motor Dysfunction
Source: Front Neurol. 2020 Sep 18;11:886. doi: 10.3389/fneur.2020.00886 (PMC7530842; doi:10.3389/fneur.2020.00886)
Supplement: Supplementary Table 1 — Stimulation parameters of patients with PD with DBS. Electrodes were targeted to stimulate either the subthalamic nucleus (STN) or the globus pallidus internus (GPi). [file Table_1.pdf]

		Voltage		Pulse Width (µS)		Stimulation Frequency (Hz)	
**Patient**	**Location**	**L**	**R**	**L**	**R**	**L**	**R**
s0004SB	STN	3.3	3.3	60	60	130	130
s0015DE	STN	3	2.5	60	60	135	135
s0023AT	STN	3.2	2.5	60	60	130	130
s0097GW	STN	2	2.2	60	60	130	130
s0099WL	STN	2	3.3	90	90	130	130
s0101AQ	GPi	1.5	2.2	90	90	130	130
s0102WX	STN	1	1.7	90	90	130	130
s0103KB	GPi	3.2	2.5	90	90	130	130

Supplementary Table 1 – *Stimulation parameters of patients with PD with DBS. Electrodes were targeted to stimulate either the subthalamic nucleus (STN) or the globus pallidus internus (GPi).*
